# Intravenous postoperative fluid prescriptions for children: A survey of practice

**DOI:** 10.1186/1471-2482-8-10

**Published:** 2008-06-09

**Authors:** Polly Davies, Tim Hall, Tariq Ali, Kokila Lakhoo

**Affiliations:** 1 Department of Anaesthetics, Northampton General Hospital, Northampton, UK; 2 Department of Paediatric Surgery, Addenbrookes Hospital, Hills Rd, Cambridge, CB2 0QQ, UK; 3 Nuffield Department of Anaesthetics, John Radcliffe Hospital, Oxford, UK; 4 Department of Paediatric Surgery, John Radcliffe Hospital, Oxford, UK

## Abstract

**Background:**

Postoperative deaths and neurological injury have resulted from hyponatraemia associated with the use of hypotonic saline solutions following surgery. We aimed to determine the rates and types of intravenous fluids being prescribed postoperatively for children in the UK.

**Methods:**

A questionnaire was sent to members of the British Association of Paediatric Surgeons (BAPS) and Association of Paediatric Anaesthetists of Great Britain and Ireland (APAGBI) based at UK paediatric centres. Respondents were asked to prescribe postoperative fluids for scenarios involving children of different ages. The study period was between May 2006 and November 2006.

**Results:**

The most frequently used solution was sodium chloride 0.45% with glucose 5% although one quarter of respondents still used sodium chloride 0.18% with glucose 4%. Isotonic fluids were used by 41% of anaesthetists and 9.8% of surgeons for the older child, but fewer for infants. Standard maintenance rates or greater were prescribed by over 80% of respondents.

**Conclusion:**

Most doctors said they would prescribe hypotonic fluids at volumes equal to or greater than traditional maintenance rates at the time of the survey. A survey to describe practice since publication of National Patient Safety Agency (NPSA) recommendations is required.

## Background

Several publications in the last decade describe the syndrome of hospital acquired hyponatraemia in children [1-3]. The usual causal factor is the administration of hypotonic saline solutions [4]. Readily available hypotonic fluids in the UK include sodium chloride 0.18% with glucose 4% and sodium chloride 0.45% with glucose 5% which respectively have one fifth and one half the tonicity of plasma.

The danger of hyponatraemia is cerebral oedema resulting in seizures, neurological deterioration and sometimes in tentorial herniation and death. This can cause permanent neurological injury and death [5]. Hyponatraemia occurs as the hypotonic saline is a source of free water, which cannot be excreted if Anti Diuretic Hormone (ADH) levels are elevated due to the surgical stress response. The mechanism of action of ADH is to impair free water excretion.

Following a hyponatraemic death associated with the use of sodium chloride 0.18% with glucose 4% in a child with gastroenteritis, the Royal College of Anaesthetists issued a bulletin following a request from Royal College of Paediatrics and Child Heath, advising caution with its use [6]. As the post operative child is particularly at risk [4,6,7], we surveyed the current practice of postoperative intravenous fluid prescription by Paediatric surgeons and Paediatric anaesthetists.

## Methods

A postal questionnaire was sent to members of the BAPS and APAGBI working at tertiary referral centres in the UK. The study period was between May and November 2006. The questionnaire asked the respondent to document the type and volume of postoperative fluids prescribed in the following clinical scenarios.

1. An elective excision of an abdominal duplication cyst in a 6 kg, six-month-old baby.

2. A 28 kg, nine-year-old child following an uncomplicated open appendicectomy.

3. A 9 kg, nine month old infant following an urgent laparotomy for an intussusception.

The questionnaire stated that all three children were well perfused and haemodynamically stable at the end of surgery. For each case the participant was asked what type of fluid they would prescribe for the first 24 hours postoperatively, and at what infusion rate.

Standard maintenance rates were defined as 100 mls per kilogram for the first 10 kilograms of body weight, 50 mls per kg for the next 10 kg and 25 mls per kg thereafter per 24 hours [8] corresponding to 4, 2 and 1 ml per kilogram per hour respectively. Space was provided on the questionnaire to detail any other orders or observations that the respondent would add to the prescription chart. A stamped addressed envelope was included.

## Results

### Response

A total of 364 questionnaires were sent. We had a 51% response rate from 120 forms sent to Paediatric surgeons and a 47% response rate from 244 forms sent to anaesthetists. We excluded 15 questionnaires from anaesthetists which had not been completed – the respondents stating that in their institution surgeons wrote or altered all postoperative fluid orders. Prescription orders varied widely. Most respondents used commercially available solutions although 3% made up their own fluid by adding dextrose to standard pre-prepared solutions.

### Fluid Type

The type of fluid prescribed is shown in table 1. The most frequently used solution by both groups of respondents for both cases was sodium chloride 0.45% with glucose 5%. Hypotonic sodium chloride 0.18% with glocose4% was prescribed by 20–25% of anaesthetists and 36–39% of paediatric surgeons, depending on the clinical scenario described. Isotonic fluids (normal saline or Hartmanns solution) were prescribed by 41% of anaesthetists and 10% of surgeons in the older child. Only 8% of anaesthetists and 1% of surgeons prescribed isotonic solutions for the younger child having elective surgery.

**Table 1:** Type of fluid used by anaesthetists and surgeons for three post operative scenarios in children

Type of fluid	Case 1.	Case 1	Case 2	Case 2	Case 3	Case 3
	Anaesthetists	Surgeons	Anaesthetists	Surgeons	Anaesthetists	Surgeons

4% dex/0.18% saline	25/100 (25%)	22/61 (36.1%)	24/100 (24%)	23/61 (37.7%)	20/100 (20%)	24/61 (39.3%)
10% dex/0.18% saline	1/100 (1%)	4/61 (6.5%)	0	0	0	3/61 (4.9%)
2.5% dex/0.45% saline	7/100 (7%)	0	8/100 (8%)	1/61 (1.7%)	8/100 (8%)	1/61 (1.7%)
5% dex/0.45% saline	35/100 (35%)	29/61 (47.5%)	24/100 (25%)	31/61 (50.8%)	33/100 (33%)	29/61 (47.5%)
10% dex/0.45% saline	1/100 (1%)	1/61 (1.7%)	0	0	1/100 (1%)	1/61 (1.7%)
5% dex/1/2 Hartmanns	2/100 (2%)	0	2/100 (2%)	0	2/100 (2%)	0
0.9% saline	1/100 (1%)	0	18/100 (18%)	3/61 (4.9%)	3/100 (3%)	1/61 (1.7%)
Hartmanns	2/100 (2%)	1/61 (1.7%)	23/100 (23%)	2/61 (3.2%)	15/100 (15%)	2/61 (3.3%)
1% dex/Hartmanns	3/100 (3%)	0	0	0	1/100 (1%)	0
5% dex/0.9% saline	2/100 (2%)	0	0	1/61 (1.7%)	1/100 (1%)	0
10% dex/Hartmanns	0	0	0	0	1/100 (1%)	0
Total answering	88/100 (88%)	59/61 (96.7%)	100/100 (100%)	61/61 (100%)	86/100 (86%)	61/61 (100%)
Left unanswered	12/100 (12%)	2/61 (3.3%)	0	0	14/100 (14%)	0

Overall, standard maintenance rates were prescribed by 63% of anaesthetists and surgeons as shown in table 2. Fewer than 20% of surgeons and anaesthetists use volumes less than standard maintenance. Between 8–35% of respondents prescribed volumes that were greater than maintenance rates. 5% (5/100) of anaesthetists and 16% (10/61) surgeons calculated maintenance rates incorrectly for the older child.

**Table 2:** Volume of fluid used by anaesthetists and surgeons for three hypothetical post operative scenarios in children

Volume of fluid	Case 1	Case 1	Case 2	Case 2	Case 3	Case 3
	Anaesthetists	Surgeons	Anaesthetists	Surgeons	Anaesthetists	Surgeons

50–74% of maintenance	13/100 (13%)	6/61 (9.8%)	8/100 (8%)	1/61 (1.7%)	11/100 (11%)	5/61 (8.2%)
76–99% of maintenance	2/100 (2%)	6/61 (9.8%)	11/100 (11%)	11/61 (18%)	5/100 (5%)	7/61 (11.4%)
100% maintenance	65/100 (65%)	42/61 (68.8%)	65/100 (65%)	25/61 (40.1%)	55/100 (55%)	43/61 (70.4%)
> 100% maintenance	8/100 (8%)	6/61 (9.8%)	16/100 (16%)	23/61 (38.3%)	15/100 (15%)	5/61 (8.2%)
Total answering	88/100 (88%)	60/61 (98.3%)	100/100 (100%)	60/61 (98.3%)	86/100 (86%)	60/61 (98.3%)
Left unanswered	12/100 (12%)	1/61 (1.7%)	0	1/61 (1.7%)	14/100 (14%)	1/61 (1.7%)

## Discussion

Despite widespread publicised concerns, over 20% of anaesthetists and 38% of surgeons still prescribe the very hypotonic sodium chloride 0.18% with glucose 4%. This is putting large numbers of children at risk of hyponatraemia and its devastating consequences. However with recent NPSA guidelines and trust actions of withdrawal of all sodium chloride 0.18% solution, this should be avoided.

Most prescriptions were for standard maintenance rates based on the original work of Holliday and Segar almost 50 years ago [8]. Their calculations were based on calorie requirements and calculated from body weight. Although the action of ADH had been described at that time, it was not known then that surgery was a potent trigger for its release. There is now good evidence that these standard maintenance rates significantly overestimate postoperative fluid requirements by up to 50% [4,9-12]. In our survey fewer than 20% of prescriptions were for volumes less than standard maintenance.

The scenarios chosen were typical for a tertiary care paediatric surgical department and were uncomplicated to avoid complex answers. Abdominal cases were chosen as other types of paediatric surgery do not necessarily require postoperative fluids. Postoperative fluid therapy prescriptions are often written according to a departmental policy. We therefore only asked what fluids are *typically *prescribed and at what rate.

More difficult scenarios may have prompted more complex and individually tailored prescriptions that would have obscured our objectives. Minimal blood loss would be anticipated for all cases, but the urgency of surgery and potential for on-going third space and gastrointestinal losses varied. This was to determine if anticipated losses prompted a different fluid prescription both in tonicity and quantity.

The simplified nature of the questionnaire did not allow the patient to be regularly assessed and treatment tailored accordingly. This would have reflected actual practices more accurately. However, with this in mind space was allocated on the questionnaire for further comments. Most respondents who provided additional information detailed the importance of regular review, monitoring of haemodynamic status and blood glucose. Only four mentioned sodium specifically. Reassuringly all fluid boluses prescribed to replace nasogastric losses or to improve perfusion were with isotonic solutions.

Worryingly between 8 and 38.8% of respondents gave maintenance fluid at volumes far greater than standard rates, presumably to compensate for blood loss, third space or gastrointestinal losses. As these losses are isotonic and the maintenance fluid was usually hypotonic, this practice is particularly dangerous.

Also alarming is the apparent inability of some respondents to recall the maintenance formula correctly. 5% of anaesthetists and 18% of surgeons multiplied the entire weight by 100 mls/kg/day. This was of no consequence for the younger children who were less than 10 kg, but grossly overestimated the volume of fluid required for the 28 kg child. Such errors put children further at risk of hyponatraemia. The heavier the child, the more significant this error becomes.

Given the warning from two Royal Colleges and widespread published concerns from a number of authors calling for the use of sodium chloride 0.18% with glucose 4% to be revised [1-4,6,13-17], we were surprised at the number of respondents who said they would use the solution. However the solution has now been withdrawn from general ward areas and theatres. Isotonic solutions have been suggested as more appropriate [1,4,5,7,13-17], yet few anaesthetists and surgeons said they are using them, particularly in younger children. This may be because the potential to precipitate hypernatraemia has been highlighted [9].

Those concerned argue that hypotonic fluids are appropriate, but at lower rates of infusion [9,11,12]. Whether hyponatraemia is primarily due to the tonicity of the fluid or the rate of infusion has not been clarified. [11,12,14,16]. There is, however, agreement that sodium chloride 0.18% solutions at standard maintenance rates are unacceptable [1-7,9-16]. A consensus between anaesthetists, surgeons and paediatricians is required to establish optimal postoperative fluid therapy. In particular, the rate of standard maintenance infusions, and the type of solution. This consensus may now be achieved due to the clear guidance of the NPSA [18].

## Conclusion

Based on current evidence and NPSA guidelines sodium chloride 0.18% solutions should be abandoned as a maintenance fluid for children in the post operative period.

Children with anticipated additional fluid losses should have them replaced with isotonic solutions only (sodium chloride 0.9% or Hartmans). Those at particular risk of hypoglycaemia (e.g. small neonates, excessive starvation) should receive glucose containing solutions. All fluid boluses should be 20 ml/kg of isotonic fluid or 2 aliquots of 10 ml/kg over a short time period. As there is no 'one size fits all' prescription, the importance of regular review with electrolyte monitoring cannot be overestimated.

## Competing interests

The authors declare that they have no competing interests.

## Authors' contributions

PD participated in the data analysis and drafted the manuscript. TH carried out the postal survey, participated in data analysis and helped to draft the manuscript. TA conceived of the study, participated in its design, statistical analysis and coordination. KL conceived of the study, participated in its design and was the main coordinator. All authors read and approved the final manuscript.

## Pre-publication history

The pre-publication history for this paper can be accessed here:


